# Profiling helper T cell subset gene expression in deer mice

**DOI:** 10.1186/1471-2172-7-18

**Published:** 2006-08-17

**Authors:** Lauren Oko, Bethany Aduddell-Swope, Derall Willis, Robyn Hamor, Teresa A Coons, Brian Hjelle, Tony Schountz

**Affiliations:** 1 School of Biological Sciences, University of Northern Colorado, 1556 Ross Hall, Greeley, CO 80639, USA; 2 Department of Biology, Mesa State College, 1100 North Ave., Grand Junction, CO 81501, USA; 3 Saccomanno Research Institute, 2530 N. 8^th ^Street, Wellington Bldg. 4, Ste. 100, Grand Junction, CO 81501, USA; 4 Center for Infectious Diseases and Immunity, Departments of Pathology, Biology, and Molecular Genetics & Microbiology, University of New Mexico School of Medicine, Albuquerque, NM 87131, USA

## Abstract

**Background:**

Deer mice (*Peromyscus maniculatus*) are the most common mammals in North America and are reservoirs for several zoonotic agents, including Sin Nombre virus (SNV), the principal etiologic agent of hantavirus cardiopulmonary syndrome (HCPS) in North America. Unlike human HCPS patients, SNV-infected deer mice show no overt pathological symptoms, despite the presence of virus in the lungs. A neutralizing IgG antibody response occurs, but the virus establishes a persistent infection. Limitations of detailed analysis of deer mouse immune responses to SNV are the lack of reagents and methods for evaluating such responses.

**Results:**

We developed real-time PCR-based detection assays for several immune-related transcription factor and cytokine genes from deer mice that permit the profiling of CD4^+ ^helper T cells, including markers of Th1 cells (T-bet, STAT4, IFNγ, TNF, LT), Th2 cells (GATA-3, STAT6, IL-4, IL-5) and regulatory T cells (Fox-p3, IL-10, TGFβ1). These assays compare the expression of in vitro antigen-stimulated and unstimulated T cells from individual deer mice.

**Conclusion:**

We developed molecular methods for profiling immune gene expression in deer mice, including a multiplexed real-time PCR assay for assessing expression of several cytokine and transcription factor genes. These assays should be useful for characterizing the immune responses of experimentally- and naturally-infected deer mice.

## Background

Deer mice (*Peromyscus maniculatus*) are the principal hosts of Sin Nombre virus (SNV), which causes the great majority of hantavirus cardiopulmonary syndrome (HCPS) cases in North America [1-3]. Despite a neutralizing antibody response, deer mice become persistently-infected with SNV without discernible pathology and can shed virus in excrement [4-6]. The mechanism by which SNV evades a sterilizing immune response in deer mice is unknown.

SNV principally infects capillary endothelial cells in humans and deer mice without conspicuous cytopathic effects [4,7]. Immunochemical evaluation of lung tissues from humans and deer mice reveals the presence of viral antigens; however, no pulmonary inflammation is observed in deer mouse lungs. In addition, HCPS patients, but not deer mice, have mononuclear infiltrates in their lungs. These cells produce several proinflammatory cytokines, including IL-1β, IL-2, IL-4, IFNγ, TNF and lymphotoxin-α (LT) [8-10]. Isolation of SNV-specific human T cells suggests Th1- and Tc1-mediated immune responses in such patients. Because of the absence of cytopathology, it is thought that the etiologic mechanism of HCPS is principally a cytokine-mediated immunopathology.

Deer mice are divergent from the common laboratory house mouse (*Mus musculus*) and rat (*Rattus norvegicus*) by 25 million years [11]. This substantial divergence has led to variations that render most immunological reagents for these species inadequate for evaluating deer mouse immune responses [12]. Because of this, methods for profiling T cell gene expression and for evaluating cytokine responses in deer mice must be developed in order to assess such responses during the course of infection with SNV.

Conventional antibody-based methods for quantitative cytokine detection rely upon the generation of pairs of monoclonal antibodies to distinct epitopes for use in capture ELISAs. These assays usually require the cloning of full-length cDNAs for each cytokine, expression and production of recombinant cytokines, and production of monoclonal antibodies. This process requires substantial effort, expertise and expense.

The development of real-time PCR methods to detect gene expression has resulted in the rapid development of many gene expression assays. One such method for detecting cytokines from unusual species employs the DNA-intercalating dye SYBR Green I [13-16], which fluoresces when bound to double-stranded DNA. In addition, these assays are readily multiplexed from small quantities of cDNA.

Unlike the production of monoclonal antibodies, the development of real-time PCR assays to detect gene expression requires only partial cDNA sequence data, and we recently cloned many such deer mouse sequences [17-19]. Using these sequences, we have developed real-time PCR assays that may useful for evaluating T cell subset responses in deer mice, including Th1, Th2 and regulatory T (Treg) cells [20-29]. In addition, we have developed conventional PCR detection assays for the expression of the subset-specific transcription factors, T-bet, GATA-3, Fox-p3, STAT4, and STAT6. Together, these assays may allow the discrimination of helper T cell subsets in deer mice.

## Results

### Generation of KLH-specific T cell lines

We previously described methods for long-term culture of outbred deer mouse T cell lines using autologous bone marrow-derived antigen presenting cells [30]. Our current efforts describe a quantitative means of determining transcription factor and cytokine gene expression in such T cell lines using PCR. Polyclonal lymph node-derived CD4^+ ^T cell lines from two deer mice, DM21 and DM22, were established and evaluated for their proliferative capacity upon recall challenge with antigen. The lines' proliferative characteristics were similar to our previous results, with half-maximal proliferation at about 1 μg/ml of KLH [30].

### Detection of transcription factor gene expression

We developed multiplex a real-time PCR detection assay for Th1, Th2 and Treg transcription factors and cytokines based upon the use of SYBR Green I DNA-binding fluorochrome. This assay determines the relative change in gene expression by comparing identical T cell/APC cultures with or without antigen exposure after 42 hours. This approach allowed us to determine the relative template abundance (RTA) induced in T cells that are activated by antigen.

Based upon the half-maximal proliferative capacity, bulk cultures of T cells and autologous APC were established with or without 1 μg/ml of KLH and incubated for 42 hours to allow antigen processing and presentation to T cells. Total RNA was extracted for assessment of transcription factor gene expression by real-time PCR of cDNA (Figure 2). Concanavalin A-activated deer mouse splenocytes served as a control. We detected the expression of each gene using real-time PCR. We obtained adjusted cycle threshold (CT) means of triplicate samples by subtracting the instrument-determined CT values from our total cycle number of 50 for KLH-stimulated and unstimulated T cell lines (Figure 2A). The ratio of expression of KLH-stimulated and unstimulated cultures was then calculated to determine the RTA for each T cell line (Figure 2B).

**Figure 1:**
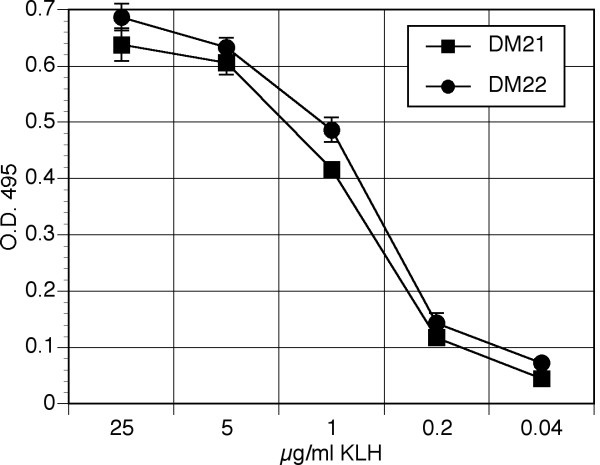
**Proliferation of KLH-specific deer mouse T cells**. Deer mice were immunized with KLH and T cell lines were established from draining lymph nodes. T cells, autologous bone marrow-derived APC and KLH were cultured for 72 hours and proliferation was determined by MTS assay. Each point represents the mean of duplicate samples with error bars representing the standard deviation.

**Figure 2:**
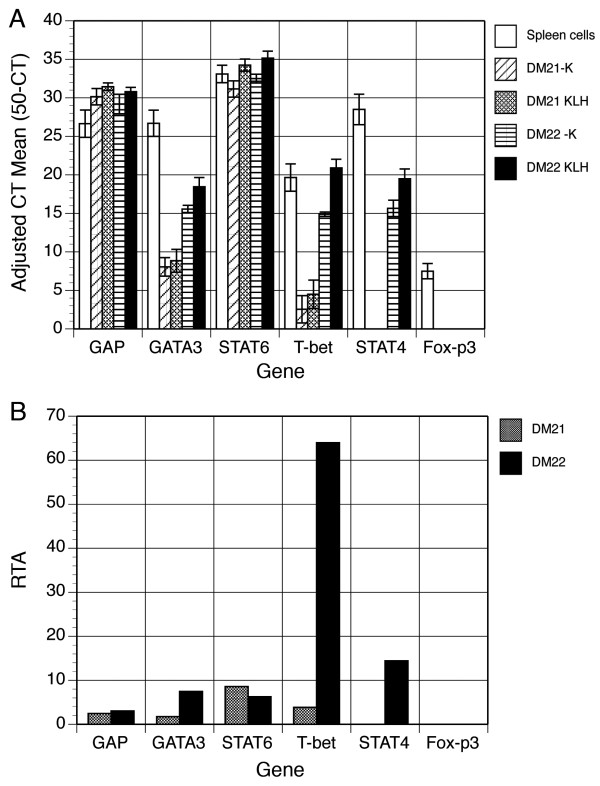
**Real-time PCR detection of deer mouse transcription factors**. Total RNA was extracted from 42 hour cultures of T cells with or without KLH activation and used for cDNA synthesis. Real-time PCR was performed using primers described in Table 1 to detect the expression of transcription factors associated with Th1, Th2 or Treg T cells. The mean CT values and standard deviations from triplicate samples obtained from the instrument were subtracted from the number of cycles performed (50) to determine adjusted CT means for unstimulated (-K) and stimulated (KLH) T cell cultures (A). The adjusted CT mean values were then used to calculated the cycle difference (CD) by subtracting the -K value from the KLH value. The relative template abundance (RTA) was then determined by calculating the log_2 _value of the CD (2^CD^) (B).

Both T cell lines constitutively expressed Th2-specific GATA3 at low to moderate levels; however, substantial increase in antigen-induced expression occurred only with the DM22 line. STAT6 was abundantly expressed by both lines with increased expression in the presence of antigen. Th1-specific T-bet was expressed at low levels in DM21, but at high levels in DM22. Its expression was dramatically increased in DM22 T cells cultured with KLH. DM21 did not express the Th1 transcription factor STAT4, while DM22 expressed it in both antigen-stimulated and unstimulated cells. Both lines expressed the Th2 transcription factor STAT6. Treg-specific Fox-p3 was not detected in either line, although it was expressed at very low levels by activated splenocytes.

### Detection of cytokine gene expression

**Figure 3:**
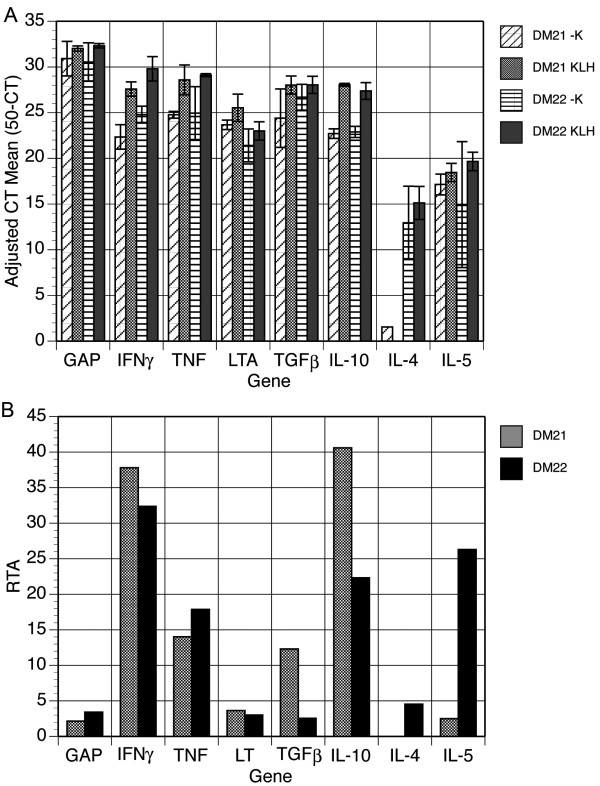
**Real-time PCR detection of deer mouse cytokine gene expression**. Template cDNA was from RNA samples described in Figure 2. Real-time PCR was performed using primers described in Table 1. Adjusted mean CT values for both antigen-stimulated and unstimulated cells (A) and the calculated RTA values for each cytokine cDNA (B).

Antigen activation of the DM21 T cell line resulted in abundant expression levels of IFNγ and IL-10, as well as moderate levels of TNF and TGFβ1, but there was little change in expression of LT or IL-5 (Figure 3). IL-4 expression was not detected in these cells. The DM21 line expressed abundant IFNγ, IL-5 and IL-10, moderate levels of TNF, but little TGFβ1, LT or IL-4.

## Discussion

Deer mice and related peromyscine species are important reservoirs for SNV and other human pathogens [31-39]. Although Syrian golden hamsters can be experimentally-infected with SNV [40], only humans, deer mice and, occasionally, other rodents are known to be naturally susceptible to SNV, with dramatically different outcomes. Spillover to related rodent species does occur, such as in pinyon mice (*P. truei*) [41] but such transmission does not appear to be an important mode of SNV maintenance. The species-specificity and apathogenic infection of hantaviruses for their rodent hosts are well-documented [3,4], but poorly understood. It is evident that such relationships have evolved over millions of years of coadaptation, as the rodents and their hantaviruses have undergone radial divergence.

Many viruses evade sterilizing immune responses with virally-encoded proteins that modulate the host response in a manner favorable to the virus. These viruses typically have many genes; however, hantaviruses are relatively simple, encoding four polypeptides, none of which have been shown to possess immunomodulatory activities. Given that deer mice produce high-affinity neutralizing IgG to SNV [4,5,34,42] an adaptive T cell response must occur during infection, since these events (affinity maturation and class switching) are mediated by helper T cells and their cytokines. Few reagents for laboratory mouse immune markers and cytokines are cross-reactive with deer mouse proteins ([12] and unpublished observations), thus it has become necessary to develop novel tools for examining T cell responses in deer mice. We previously generated sequence data for many deer mouse immune-function genes [17,19] and present here their utility for assessing responses of antigen-specific helper T cells.

Several functional helper T cell subsets in the laboratory mouse have been characterized based on the expression of clone-specific transcription factors and cytokines [20,24-29]. Th1 cells that promote inflammation express the transcription factors STAT4 and T-bet, and the cytokines IFNγ, TNF and LT. Th2 cells, which augment antibody synthesis, express STAT6 and GATA3, and IL-4 and IL-5. Treg cells are apparently a heterogeneous group of potent anti-inflammatory cells that exhibit differential expression of the transcription factor Fox-p3 and the cytokines TGFβ1 and IL-10. A fourth subset, Th17 cells, has recently been described that expresses the proinflammatory cytokine IL-17 during some autoimmune diseases [43-45], but has not been associated with infectious diseases. We sought to develop assays that would allow us to discriminate these subpopulations in the deer mouse. Although we have cloned a partial cDNA of deer mouse IL-17 (AY426970) it has insufficient sequence information for design of suitable real-time PCR primers. Thus, we focused on the discrimination of Th1, Th2 and Treg cells and our results demonstrate that we are able to detect the expression of these cytokine and transcription factor genes by real-time PCR.

We detected the expression of these genes from deer mouse cells, but we were unable to discriminate discrete subpopulations that correlate with those of the house mouse. Because the T cell lines are polyclonal, there may be distinct Th1, Th2 and Treg clones in the cultures that express the hallmark cytokines. Alternatively, the distinct characteristics used for identifying these T cells in highly inbred laboratory mice may not apply to outbred deer mice. Additional work will be required to clone deer mouse T cells in order for such characterizations to be made.

An important caveat of all gene expression assays is that some genes are translationally regulated, thus data from such assays must be evaluated with caution. It would be ideal if the presence or absence of cytokines could be determined using protein-detection assays, such as capture ELISAs. No such assays are available for the deer mouse model, so gene expression assays currently are the only option. Nonetheless, differential expression of cytokine transcripts in antigen-stimulated versus unstimulated cells is suggestive of cytokine production and secretion.

Considering the relative ease of cloning genes and developing real-time PCR assays, additional genes can be cloned and used to further characterize deer mouse immune responses, thus clarifying how rodent reservoirs and hantaviruses have evolved an immunological *détente*. This may also provide clues as to how other viruses persist in their reservoirs.

The availability of these assays will permit the characterization of immune responses in deer mice acutely or persistently infected with SNV or with other zoonotic agents. Considering that HCPS is, at least in part, caused by a cytokine-mediated immunopathologic process, understanding how deer mice remain infected with SNV without attendant pathology may suggest potential targets of therapeutic intervention for HCPS patients.

## Conclusion

We developed detection assays to assess helper T cell functions in the deer mouse. These assays will provide tools to allow extensive characterization of nonpathogenic immune responses occurring in SNV-infected deer mice and provide a rationale for studies of other host-pathogen interactions. Such characterizations may clarify the role of the pathological immune response in patients with hantavirus infections and aid in identifying potential therapeutic targets of intervention.

## Methods

### Deer mice

All methods were approved by the UNC Institutional Animal Care and Use Committee and were conducted in accordance with the Animal Welfare Act. Fifteen week-old deer mice were immunized subcutaneously with KLH emulsified in complete Freund's adjuvant as previously described [30]. After 10 days, deer mice were euthanized and inguinal lymph nodes, spleens and bone marrow were harvested.

### Cloning of transcription factor cDNAs

Primers from highly conserved regions of orthologous T-bet, GATA-3, Fox-p3, STAT4 and STAT6 cDNA sequences were used to amplify deer mouse genes using the PCR Core kit with its Q-solution (Qiagen, Valencia, CA). Amplified products were cloned into the pGEM-T Easy vector (Promega, Madison, WI) and transformed into NovaBlue cells (Novagen, Madison, WI). Plasmids were purified (Qiagen Mini-Prep kit) and sequenced (Big-Dye Sequencing kit, Applied Biosystems, Foster City, CA). BLAST was conducted to verify gene identity.

### T cell culture

T cells were produced and maintained as previously described [30]. Spleens from immunized deer mice were made into single-celled suspensions, aliquotted (10^6^/0.5 ml/vial) in 5% deer mouse medium (DMM; RPMI-1640, 315 mOsm, 50 μM β-mercaptoethanol) in 10% DMSO and stored at -70°C. Bone marrow cells were washed in 5% FBS-DMM, aliquotted (5 × 10^5^/0.25 ml/vial) and stored at -70°C. Lymph node cells were cultured with 20 μg/ml of KLH in 48 well plates for 4 days, then restimulated in 48 well plates with fresh KLH and 10^6 ^freshly-thawed and once washed autologous splenocytes. The cultures were fed at 2-day intervals by replacement with 5% FBS-DMM containing 10 ng/ml recombinant human IL-2 (R&D Systems, Minneapolis, MN). Cells were split 1:2 as wells approached confluence. After two weeks of restimulation, T cells were collected and washed, then cultured with fresh KLH and bone marrow-derived antigen presenting cells (APC; see below) for continued in vitro adaptation and expansion. The cells expressed CD4 mRNA (GenBank DQ836358) by reverse transcription PCR using introns-spanning primers (forward, 5'-GCTTGCGGAGTTTTCCTTCC-3'; reverse, 5'-CACAGCGTTGTCTTTCTGAGCC-3').

### Generation of bone marrow-derived APC

One vial of bone marrow cells from each deer mouse was quick-thawed and plated onto 35 mm bacterial Petri dishes and cultured in a final volume of 3 ml of APC medium (10% FBS-DMM supplemented with 10 ng/ml recombinant mouse GM-CSF, R&D Systems). At 2 or 3 day intervals, half of the medium was removed and replaced with fresh APC medium for 10 to 14 days, at which time large numbers of competent APCs were generated for use in T cell stimulation experiments.

### T cell proliferation assay

KLH, 2 × 10^5 ^T cells and 10^4 ^BM-APC were cultured in 5% FBS-DMM in 96-well plates for 72 hours under 7% CO_2_, then proliferation assessed by MTS assay (CellTiter-96 Aq, Promega, Madison, WI). Means and standard deviations were calculated from duplicate samples with the -K (no KLH) subtracted from each mean.

### Real-time PCR

For antigen recall experiments, 1 μg/ml KLH, 5 × 10^5 ^T cells and 5 × 10^4 ^BM-APC were cultured as above in 48 well plates for 42 hours, then total RNA was extracted (VersaGene RNA Cell kit, Gentra Systems, Minneapolis, MN). Messenger RNA was reverse-transcribed (iScript, Bio-Rad, Hercules, CA) and used as template cDNA. Primers (Table 1) were designed according to Peinnequin et al. [15] using MacVector software. Real-time PCR was performed in triplicate in 25 μl volumes using iQ SYBR Green kit (Bio-Rad) for 50 cycles with a MyiQ real-time thermal cycler (Bio-Rad) with 95°C melting (30 sec), 58°C annealing (30 sec), and 72°C extension (30 sec). Melt curve analysis was conducted to verify single products (data not shown). The relative template abundance (RTA) was determined by comparing gene expression in KLH-stimulated and unstimulated T cell cultures. First, CT means were subtracted from 50 to produce adjusted CT means and standard deviations from triplicate samples. The adjusted mean of each -K sample was subtracted from the adjusted mean of the antigen-stimulated sample to provide the cycle difference (CD). Finally, the RTA was determined by calculating 2^CD^.

**Table 1:** Primers for Real-Time PCR^1^

Gene	Forward	Reverse	Size (bp)	Acc#
GAPDH	GGTGCCAAAAGGGTCATCATCTC	GCAGGAAGCGTTGCTGATAATCTTG	114	AY841947
IFNγ	GGCTATTTCTGGCTGTTACTGCC	ATCCCCGACATCTGAGCTACTTG	94	AY289494
TNF	TGTAGCCCACGTTGTAGCAAACC	CTGGTTGTCTTTGAGATCCATGC	106	AF307013
LTA	ATGGTGTCTCCCATCTACACTTCAG	TTGAAACGGTCAGCATGGAGG	115	AF348259
IL-4	CCCCGTGCTTGAAGAACAATTC	GGACTCATTCCCAGTACAGCTTTTC	104	DQ446203
IL-5	GAAGAATCAAACTGTCCGTGGG	ACACTGCTCTTTTTGGCGGTC	94	AY843530
TGFβ1	CGTGGAACTCTACCAGAAATACAGC	TCAAAAGACAACCACTCAGGCG	96	AY455973
IL-10	TAAGGGTTACCTGGGTTGCCAAG	CAAATGCTCCTTGATTTCTGGGC	106	AF307012
GATA3	AGTCCGCATCTCTTCACCTTCC	GGCACTCTTTCTCATCTTGCCG	112	AY325113
T-bet	GATCATCACTAAGCAAGGACGGC	AGACCACATCCACAAACATCCTG	101	AY271903
Fox-p3	AAGCAGATCACCTCCTGGATGAG	TAGCACCCAGCTTCTCCTTTTCC	114	AY841945
STAT4	AACCATTTACCTTCTGGACCTGG	TTGCTCACGAAGCCCATGATGTACC	103	AY455975
STAT6	CGCTTTAGCGACTCTGAGATTGG	TCTTTGGCAGAAAATGGCTGG	104	DQ836357

^1^All sequences are listed 5' to 3'.

## Abbreviations

HCPS, hantavirus cardiopulmonary syndrome; APC, antigen presenting cell; SNV, Sin Nombre virus; LT, lymphotoxin; DMM, deer mouse medium; CFA, complete Freund's adjuvant; CT, cycle threshold; CD, cycle difference; RTA, relative template abundance; MTS, 3-(4,5-dimethylthiazol-2-yl)-5-(3-carboxymethoxyphenyl)-2-(4-sulfophenyl)-2H-tetrazolium.

## Authors' contributions

LO conducted RNA extractions and real-time PCR of antigen-specific T cells. BAS cloned and sequenced the Fox-p3, and GAP genes. DGW and TAC developed the cytokine real-time assays. RH generated and maintained bone marrow-derived APC.

BH provided IL-4 and IL-5 sequences for primer design, and contributed to the conceptual framework. TS generated T cell lines, transcription factor PCR and conceptual framework.

## References

[B1] Hughes JM, Peters CJ, Cohen ML, Mahy BW (1993). Hantavirus pulmonary syndrome: an emerging infectious disease. Science.

[B2] Nichol ST, Spiropoulou CF, Morzunov S, Rollin PE, Ksiazek TG, Feldmann H, Sanchez A, Childs J, Zaki S, Peters CJ (1993). Genetic identification of a hantavirus associated with an outbreak of acute respiratory illness. Science.

[B3] Schmaljohn C, Hjelle B (1997). Hantaviruses: a global disease problem. Emerg Infect Dis.

[B4] Botten J, Mirowsky K, Kusewitt D, Bharadwaj M, Yee J, Ricci R, Feddersen RM, Hjelle B (2000). Experimental infection model for Sin Nombre hantavirus in the deer mouse (Peromyscus maniculatus). Proc Natl Acad Sci U S A.

[B5] Botten J, Mirowsky K, Kusewitt D, Ye C, Gottlieb K, Prescott J, Hjelle B (2003). Persistent Sin Nombre virus infection in the deer mouse (Peromyscus maniculatus) model: sites of replication and strand-specific expression.. J Virol.

[B6] Yamada T, Hjelle B, Lanzi R, Morris C, Anderson B, Jenison S (1995). Antibody responses to Four Corners hantavirus infections in the deer mouse (Peromyscus maniculatus): identification of an immunodominant region of the viral nucleocapsid protein. J Virol.

[B7] Zaki SR, Greer PW, Coffield LM, Goldsmith CS, Nolte KB, Foucar K, Feddersen RM, Zumwalt RE, Miller GL, Khan AS, Rollin P, Ksiazek T, Nichol S, Mahy BW, Peters CJ (1995). Hantavirus pulmonary syndrome. Pathogenesis of an emerging infectious disease. Am J Pathol.

[B8] Ennis FA, Cruz J, Spiropoulou CF, Waite D, Peters CJ, Nichol ST, Kariwa H, Koster FT (1997). Hantavirus pulmonary syndrome: CD8+ and CD4+ cytotoxic T lymphocytes to epitopes on Sin Nombre virus nucleocapsid protein isolated during acute illness. Virology.

[B9] Kilpatrick ED, Terajima M, Koster FT, Catalina MD, Cruz J, Ennis FA (2004). Role of specific CD8+ T cells in the severity of viral hemorrhagic fever, hantavirus pulmonary. J Immunol.

[B10] Mori M, Rothman AL, Kurane I, Montoya JM, Nolte KB, Norman JE, Waite DC, Koster FT, Ennis FA (1999). High levels of cytokine-producing cells in the lung tissues of patients with fatal hantavirus pulmonary syndrome. J Infect Dis.

[B11] Crew MD, Bates LM, Douglass CA, York JL (1996). Expressed Peromyscus maniculatus (Pema) MHC class I genes: evolutionary implications and the identification of a gene encoding a Qa1-like antigen. Immunogenetics.

[B12] Vaughn J, Schountz T (2003). Discrimination of Peromyscus maniculatus leukocytes by flow cytometry. BIOS.

[B13] Budhia S, Haring LF, McConnell I, Blacklaws BA (2006). Quantitation of ovine cytokine mRNA by real-time RT-PCR. J Immunol Methods.

[B14] Odbileg R, Konnai S, Usui T, Ohashi K, Onuma M (2005). Quantification of llama inflammatory cytokine mRNAs by real-time RT-PCR. J Vet Med Sci.

[B15] Peinnequin A, Mouret C, Birot O, Alonso A, Mathieu J, Clarencon D, Agay D, Chancerelle Y, Multon E (2004). Rat pro-inflammatory cytokine and cytokine related mRNA real-time polymerase chain reaction using SYBR green. BMC Immunol.

[B16] Konnai S, Usui T, Ohashi K, Onuma M (2003). The rapid quantitative analysis of bovine cytokine genes by real-time RT-PCR. Vet Microbiol.

[B17] Herbst MM, Prescott J, Palmer AD, Schountz T (2002). Sequence and expression analysis of deer mouse interferon-g, interleukin-10, tumor necrosis factor, and lymphotoxin a. Cytokine.

[B18] Green RM, Herbst MM, Schountz T (2004). Genomic organization of  deer mouse (Peromyscus maniculatus) tumor necrosis factor. BIOS.

[B19] Schountz T, Green R, Davenport B, Buniger A, Richens T, Root JJ, Davidson F, Calisher CH, Beaty BJ (2004). Cloning and characterization of deer mouse (Peromyscus maniculatus) cytokine and chemokine cDNAs. BMC Immunol.

[B20] Agnello D, Lankford CS, Bream J, Morinobu A, Gadina M, O'Shea JJ, Frucht DM (2003). Cytokines and transcription factors that regulate T helper cell differentiation: new players and new insights. J Clin Immunol.

[B21] Belkaid Y, Rouse BT (2005). Natural regulatory T cells in infectious disease. Nat Immunol.

[B22] Chen W, Jin W, Hardegen N, Lei KJ, Li L, Marinos N, McGrady G, Wahl SM (2003). Conversion of peripheral CD4+CD25- naive T cells to CD4+CD25+ regulatory T cells by TGF-beta induction of transcription factor Foxp3. J Exp Med.

[B23] Fiorentino DF, Bond MW, Mosmann TR (1989). Two types of mouse T helper cell. IV. Th2 clones secrete a factor that inhibits cytokine production by Th1 clones. J Exp Med.

[B24] Grusby MJ (1997). Stat4- and Stat6-deficient mice as models for manipulating T helper cell responses. Biochem Soc Trans.

[B25] Kaplan MH, Grusby MJ (1998). Regulation of T helper cell differentiation by STAT molecules. J Leukoc Biol.

[B26] Mosmann TR, Cherwinski H, Bond MW, Giedlin MA, Coffman RL (1986). Two types of murine helper T cell clone. I. profiles of lymphokine activities and secreted. J Immunol.

[B27] Rouse BT, Suvas S (2004). Regulatory cells and infectious agents: detentes cordiale and contraire. J Immunol.

[B28] Takeda K, Akira S (2000). STAT family of transcription factors in cytokine-mediated biological responses. Cytokine Growth Factor Rev.

[B29] Wurster AL, Tanaka T, Grusby MJ (2000). The biology of Stat4 and Stat6. Oncogene.

[B30] Davenport BJ, Willis DG, Prescott J, Farrell RM, Coons TA, Schountz T (2004). Generation of competent bone marrow-derived antigen presenting cells from the deer mouse (Peromyscus maniculatus). BMC Immunol.

[B31] Abbott KD, Ksiazek TG, Mills JN (1999). Long-term hantavirus persistence in rodent populations in central Arizona. Emerg Infect Dis.

[B32] Bohlman MC, Morzunov SP, Meissner J, Taylor MB, Ishibashi K, Rowe J, Levis S, Enria D, St Jeor SC (2002). Analysis of hantavirus genetic diversity in Argentina: s segment-derived phylogeny. J Virol.

[B33] Breitschwerdt EB, Kordick DL (2000). Bartonella infection in animals: carriership, reservoir potential, pathogenicity, and zoonotic potential for human infection. Clin Microbiol Rev.

[B34] Gavrilovskaya I, LaMonica R, Fay ME, Hjelle B, Schmaljohn C, Shaw R, Mackow ER (1999). New York 1 and Sin Nombre viruses are serotypically distinct viruses associated with hantavirus pulmonary syndrome. J Clin Microbiol.

[B35] Homer MJ, Aguilar-Delfin I, Telford SR, Krause PJ, Persing DH (2000). Babesiosis. Clin Microbiol Rev.

[B36] Mead DG, Ramberg FB, Besselsen DG, Mare CJ (2000). Transmission of vesicular stomatitis virus from infected to noninfected black flies co-feeding on nonviremic deer mice. Science.

[B37] Nicholson WL, Muir S, Sumner JW, Childs JE (1998). Serologic evidence of infection with Ehrlichia spp. in wild rodents (Muridae: Sigmodontinae) in the United States. J Clin Microbiol.

[B38] Perz JF, Le Blancq SM (2001). Cryptosporidium parvum infection involving novel genotypes in wildlife from lower New York State. Appl Environ Microbiol.

[B39] Ravyn MD, Kodner CB, Carter SE, Jarnefeld JL, Johnson RC (2001). Isolation of the etiologic agent of human granulocytic ehrlichiosis from the white-footed mouse (Peromyscus leucopus). J Clin Microbiol.

[B40] Hooper JW, Larsen T, Custer DM, Schmaljohn CS (2001). A lethal disease model for hantavirus pulmonary syndrome. Virology.

[B41] Calisher CH, Root JJ, Mills JN, Rowe JE, Reeder SA, Jentes ES, Wagoner K, Beaty BJ (2005). Epizootiology of Sin Nombre and El Moro Canyon hantaviruses, southeastern Colorado, 1995-2000. J Wildl Dis.

[B42] Borucki MK, Boone JD, Rowe JE, Bohlman MC, Kuhn EA, DeBaca R, St Jeor SC (2000). Role of maternal antibody in natural infection of Peromyscus maniculatus with Sin Nombre virus. J Virol.

[B43] Cua DJ, Sherlock J, Chen Y, Murphy CA, Joyce B, Seymour B, Lucian L, To W, Kwan S, Churakova T, Zurawski S, Wiekowski M, Lira SA, Gorman D, Kastelein RA, Sedgwick JD (2003). Interleukin-23 rather than interleukin-12 is the critical cytokine for autoimmune inflammation of the brain. Nature.

[B44] Langrish CL, Chen Y, Blumenschein WM, Mattson J, Basham B, Sedgwick JD, McClanahan T, Kastelein RA, Cua DJ (2005). IL-23 drives a pathogenic T cell population that induces autoimmune inflammation. J Exp Med.

[B45] Murphy CA, Langrish CL, Chen Y, Blumenschein W, McClanahan T, Kastelein RA, Sedgwick JD, Cua DJ (2003). Divergent pro- and antiinflammatory roles for IL-23 and IL-12 in joint autoimmune inflammation. J Exp Med.

